# Cross-country comparison of income-related inequality in physical functional disability among middle-aged and older adults: Evidence from 33 countries

**DOI:** 10.7189/jogh.13.04053

**Published:** 2023-05-19

**Authors:** Jiajia Li, Shiqi Lin, Xiaojin Yan, Yue Wei, Fan Yang, Lijun Pei

**Affiliations:** Institute of Population Research, China Center on Population Health and Development, Peking University, Beijing, China

## Abstract

**Background:**

Physical functional disability is prevalent among middle-aged and older adults, with substantial health inequality. This study compared cross-country variation in the prevalence and inequality of physical functional disability and investigated the potential determinants of household income-related inequality.

**Methods:**

This cross-sectional study used data from 33 countries between 2017 and 2020, containing 141 016 participants aged 55 years and older. Physical functions were grouped into three domains: activities of daily living (ADLs), instrumental activities of daily living (IADLs), and mobility function. Physical functional disability of each domain was indicated by having some difficulty with the activity. We first estimated the prevalence of physical functional disability in each country. Second, the concentration index was used to quantify household income-related health inequality. Finally, recentred influence function (RIF) decomposition method was used to decompose the inequality into individual and country-level determinants.

**Results:**

Physical functional disability prevalence was higher in lower-middle-income countries than in high-income countries and more prevalent in the poor-income groups in all study countries. Besides, health inequality in different domains of disability was higher in high-income countries than in low-income countries. Regarding determinants of health inequality, we found that individual married, tertiary education, and country-level health infrastructure and resources were associated with reduced health inequality. In contrast, age, unhealthy lifestyles, and chronic diseases were associated with increased health inequality.

**Conclusions:**

Inequality in physical functional disability among middle-aged and older adults varies substantially across countries, with individual and macro determinants being contributing factors. Policies to achieve healthy ageing and reduce the inequality of physical function disability can focus on improving individual healthy lifestyles and country health care facilities.

Physical functional disability is a global public health concern, especially among ageing populations [1]. With accelerated population ageing, it is estimated that one in six people worldwide will be 65 or older by 2050 [2]. The growing ageing population commonly encounters disease burdens and physical function disabilities [3,4]. According to United Nations (UN) estimates, more than 46% of older adults who are 60 or older experienced a disability, while more than 250 million experienced moderate to severe disabilities [5]. Alongside population ageing, increases in physical functional disability present a significant social-economic burden to the social and health care system [4]. Simultaneously, physical functional disability also vary across countries and between social-economic groups [6,7], with several factors contributing to social-economic disparities in physical functional disability. Understanding the cross-national variation in the prevalence and the inequality of physical functional disability distribution across different social-economic groups can provide global insights and evidence for planning health promotion and prevention programs.

Previous studies found substantial variations and inequality in physical functional disability. A higher prevalence of disability was found in low-middle-income countries compared to high-income countries [1,6]. As for disparity in physical function disability across individual economic levels, evidence from the USA and UK populations found that low wealth was associated with disability among older adults [8]. A trend study in Europe countries also found that older adults with low-income levels had higher rates of functional limitations than individuals with high-income levels [9]. Further, evidence from 43 low-income and middle-income countries between 2002-2004 also found that wealth levels were inversely associated with disability rates among older adults [10]. Regarding the magnitude of physical functional disability inequality, studies found substantial differences across countries, with higher inequality in high-income countries [6,7]. However, despite substantial evidence, few studies have compared and investigated the magnitude of variation and income-related inequality of physical functional disability across countries with different economic development levels [6,7]. Besides, due to the inconsistency of disability measurements and survey design across different regions, previous research has remained inconclusive [11].

Evidence on differences in physical functional disability inequality across countries indicates that country-level factors might shape the health inequality of individuals. According to the International Classification of Functioning, Disability, and Health (ICF) framework, disability is the outcome of interacting with individuals and their environment [12]. Therefore, the income-related inequality of physical functional disability is also affected by the complex interactions between individuals and their country characteristics. Previous decomposition analysis suggests that inequality is determined by individual-level explanatory variables like social-demographic characteristics, education, wealth [13,14], lifestyle [15], country-level socioeconomics, culture, and health care systems [7,16,17]. However, few studies have investigated individual and macro-level factors simultaneously, and it also remains unclear whether the association between income-related health inequality and these explanatory variables is consistent across countries.

We aimed to characterise and compare the cross-national variation in the prevalence and inequality of physical functional disability among middle-aged and older adults. Due to the cross-country variation in social-economic development inequality, we hypothesised that the prevalence and inequality in physical functional disability also follow the social-economic gradient patterns. We also decomposed income-related inequality into individual and country-level explanatory variables, and assessed whether individual-level education, lifestyle, and country-level health infrastructure and resources were associated with reduced health inequality. This study addresses gaps in current literature by providing a comprehensive assessment with harmonised measurements and using the most recent survey data from 2017 to 2020 across 33 countries with different economic development levels.

## METHODS

### Data sources

We conducted a cross-sectional analysis using secondary data from six large nationally representative longitudinal ageing cohorts encompassing 33 countries (Austria, Belgium, Bulgaria, China, Croatia, Cyprus, Czech Republic, Denmark, Estonia, Finland, France, Germany, Greece, Hungary, India, Israel, Italy, Korea, Latvia, Lithuania, Luxembourg, Malta, Mexico, the Netherlands, Poland, Romania, Slovakia, Slovenia, Spain, Sweden, Switzerland, the UK, and the USA). Specifically, The Health and Retirement Study (HRS) Wave 14 (2018-2019) of the USA, the UK Longitudinal Study of Ageing (ELSA) Wave 9 (2018-2019), the Survey of Health, Ageing and Retirement in Europe (SHARE) Wave 8 (2019-2020), the Korean Longitudinal Study of Aging (KLoSA) Wave7 (2018-2019), the Longitudinal Aging Study in India (LASI) Wave 1 (2017-2019), the Mexican Health and Aging Study (MHAS) Wave 5 (2018-2019) and the China Health and Retirement Study (CHARLS) Wave 4 (2018-2019). Previous publications have described the recruitment strategy and design of these studies in detail [18-24]. For the convenience of cross-national comparison, we utilised data from the Harmonized HRS Version C, Harmonized ELSA Version G.2, Harmonized SHARE Version F, Harmonized KLoSA Version D.2, Harmonized LASI data set Version A.2, Harmonized MHAS Version C, and Harmonized CHARLS Version D developed by the Gateway to Global Aging Data project [25], which provide internationally comparable data on aforementioned cohorts.

We selected the latest survey waves to compare information from the same period. We included only participants aged 55 years or older and excluded participants whose physical functional measurement information was missing or incomplete. Finally, we included 141 016 participants between 2017 to 2020 from 33 countries. The 33 countries were grouped into lower-middle-income, upper-middle-income, and high-income country groups according to World Bank’s 2017 classification [26].

Besides individual-level data, we combined the country-level macro information on social-economic development, health, and health care systems. Country-level data of each country in 2017 were accessed from World Bank online Databank database [27].

### Health outcomes

We categorised physical functional health measures of middle-aged and older adults into three domains: activities of daily living (ADLs), instrumental activities of daily living (IADLs), and mobility functions. We measured ADLs with a five-item summary measure, including bathing, dressing, eating, getting in/out of bed, and using the toilet, IADLs with a four-item summary measure, including managing money, taking medications, shopping for groceries, and preparing hot meals, and mobility functions with a seven-item summary measure, including walking one block or 100 yards/meters, climbing several flights of stairs, getting up from a chair after sitting for long periods, stooping, kneeling, or crouching, reaching or extending arms above shoulder level, lifting or carrying objects weighting over five kilograms or 10 pounds, picking up a small coin from the table activities. The mobility function was not available in KLoSA. Table S1 in the **Online Supplementary Document** provides a detailed description of these measurements.

Correspondingly, we defined physical functional disability by the disability status of each domain. Both ADLs, IADLs, and mobility function responses were dichotomised to those who have any difficulty in performing the tasks vs those who have no difficulty.

### Household income status

We estimated the income-related health inequalities of physical functional disability by using the household income status of each participant. Previous research has shown that total household income is a better indicator of living standards than individual income [28]. We therefore used the annual per person total household income level as an indicator of income status. Total household income consisted of a wide range of income sources for household members, including individual and spouse earnings, capital income, income from an employer or private pension and annuity, income from public pensions, other government transfers, other regular payments. The sum of the abovementioned income over the past 12 months was used to calculate the annual per capita total household income. In considering the heterogeneous development of each country, the classification of income groups was conducted separately within each country. Specifically, we categorised participants into five groups by quintile of the annual per person total household income within each country (quintiles 1 (the lowest) to quintiles 5 (the highest)).

### Explanatory variables

According to the ICF framework, household income-related health inequality in physical functional disability among middle-aged and older adults may stem from individual and contextual factors. We thus classified the potential explanatory variables of health inequality into two levels: individual-level explanatory and social-demographic variables (including age, gender, marital status, and education level (less than upper secondary, upper secondary and vocational training, and tertiary)). Lifestyle variables, including smoking and drinking behaviours, indicate whether the participants have smoked/drank or are smokers/drinkers. Self-reported chronic diseases indicate whether the respondent has been diagnosed by the doctor with any conditions, including hypertension, diabetes, cancers, chronic lung diseases, stroke, and arthritis or rheumatism.

Country-level variables included hospital beds per 1000 people, people using safely managed sanitation services, per cent of the rural population, current health expenditure, gross enrolment ratio for tertiary education, and gross domestic product (GDP). We then categorised participants into four levels by quantile of these variables (quantile 1 (the lowest) to quantile 4 (the highest)). Table S2 in the **Online Supplementary Document** provides a detailed description of these measurements.

### Statistical analysis

#### Measuring disability prevalence

To provide a comparable and representative population estimation of the disability prevalence of ADL, IADL, and mobility by country, we used the sample weights of each survey to account for the country survey sample designs by using stratum and cluster variables. We applied the post-stratification weights by using the global population age composition information from World Population Prospects (WPP) 2019 [29] to adjust for the effects of age composition across different countries. Finally, we calculated the estimated prevalence as percentages within 95% confidence intervals (95% CIs) and used the heatmap to visualise the patterns of disability prevalence for each country.

#### Measuring inequality

The concentration index is commonly used to measure health and health care inequality; it is a bivariate rank-dependent index that measures inequality in one variable over the distribution of another variable [30]. We considered the interest variable as the disability status of ADL, IADL, and mobility function, and the rank variable as household income level. However, with binary variables, the standard concentration index might violate the mirror condition since inequality in attainments does not mirror inequality in shortfalls. The generalised version of the modified concentration index by Erreygers was designed to accommodate binary health variables [31]. Thus, we used the generalised concentration index to quantify the health inequality of each country. The concentration index value ranges in (-1,1), with zero, indicating no inequality. The positive score of the concentration index indicates a pro-rich inequality of physical functional disability, ie, high-income groups tended to have disabilities more than the lower-income groups, and the negative score indicates a pro-poor inequality of physical functional disability. Besides, we also calculated the percentage difference in disability prevalence between the lowest income group (quintile 1) and the highest income group (quintile 5). We also used the bar plot to visualise the patterns of the inequality index and percentage difference for each country.

#### Decomposing inequality

The recentred influence function (RIF) regression decomposition method was used to decompose the inequality into individual and country-level determinants [32]. We first calculated the RIF of the concentration index value. Then, we adopted the two-level linear regression model to regress the RIF value of the concentration index with the potential explanatory variables from individual and country levels. Thus, based on the linear assumption, the estimated coefficients could be interpreted as an association between the potential explanatory variables and the concentration index, providing valuable information on which groups of individuals might be associated with health inequality [32].

We implemented the multivariate imputation chained equations to deal with missing values in the potential explanatory variables. All the statistical analyses were performed in Stata 17.0. Values with *P* < 0.05) were considered as statistically significant.

## RESULTS

### Prevalence of physical functional disability by characteristics

We included 141 016 participants aged 55 years or older from 33 countries enrolled between 2017 and 2020. We estimated the overall prevalence of ADL disability to be at 16.6%, (95% CI = 16.1-17.1), IADL disability at 17.6% (95% CI = 17.1-19.1), and mobility disability at 57.8% (95% CI = 57.2-58.7). Disability prevalence increased with age. Participants with physical function disability were more likely to be female, married, and have lower education and income (**Table 1**).

**Table 1:** Age-standardised-weighted prevalence of physical functional disability by characteristics*

Variables	n	ADL disability	IADL disability	Mobility disability†
**Overall**	141 016	16.5 (16.1-17.1)	17.6 (17.1-18.1)	57.8 (57.2-58.7)
**Individual level**				
Age				
*≥55*	25 539	9.0 (8.2-9.8)	10.3 (9.4-11.2)	44.6 (42.9-46.2)
*≥60*	28 477	12.1 (11.2-13.1)	15.1 (14.0-16.2)	52.8 (51.0-54.6)
*≥65*	28 146	16.5 (15.3-17.7)	19.3 (17.9-20.8)	58.8 (57.0-60.7)
*≥70*	22 389	19.5 (18.1-21.1)	22.8 (21.3-24.4)	64.6 (62.9-66.3)
*≥75*	16 578	23.8 (22.1-25.7)	27.8 (25.8-29.8)	70.4 (68.4-72.3)
*≥80*	11 460	32.4 (29.8-35.0)	39.2 (36.3-42.2)	79.4 (77.4-81.2)
*≥85*	8427	44.7 (41.5-48.0)	56.5 (53.4-59.6)	86.6 (84.4-88.6)
Gender				
*Male*	63 189	14.1 (13.4-14.8)	14.5 (13.8-15.3)	50.2 (49.0-51.3)
*Female*	77 827	18.9 (18.2-19.6)	20.4 (19.7-21.1)	65.4 (64.5-66.4)
Marital status				
*Others*	48010	18.4(17.5-19.3)	18.6(17.7-19.6)	59.2(57.8-60.5)
*Current married*	93006	15.6(15.0-16.2)	16.9(16.3-17.5)	57.1(56.2-58.0)
Education				
*Less than upper secondary*	77 494	19.6 (19.0-20.3)	22.2 (21.5-22.9)	64.5 (63.5-65.5)
*Upper secondary and vocational training*	43 627	12.1 (11.3-13.0)	10.2 (9.4-11.1)	50.2 (48.9-51.6)
*Tertiary*	19 895	7.2 (6.5-7.9)	5.9 (5.3-6.6)	39.0 (37.3-40.7)
Income level**‡**				
*Quantile 1*	29 836	23.1 (21.9-24.5)	25.3 (24.0-26.6)	68.5 (66.7-70.2)
*Quantile 2*	28 922	19.3 (18.2-20.4)	20.6 (19.4-21.7)	62.6 (61.1-64.1)
*Quantile 3*	28 227	15.8 (14.8-16.9)	17.2 (16.1-18.3)	58.7 (57.1-60.3)
*Quantile 4*	27 170	13.3 (12.3-14.4)	13.2 (12.2-14.3)	53.4 (51.7-55.2)
*Quantile 5*	26 861	11.8 (10.7-13.1)	12.3 (11.1-13.6)	49.4 (47.6-51.1)
Chronic diseases				
*No*	43 208	7.4 (6.5-8.3)	10.5 (9.5-11.5)	39.5 (38.0-41.1)
*Yes*	97 808	19.3 (18.7-19.9)	19.7 (19.1-20.3)	64.0 (63.1-64.9)
Current smoke				
*No*	121 043	17.0 (16.5-17.6)	17.7 (17.1-18.3)	59.0 (58.1-59.8)
*Yes*	19 973	14.7 (13.6-15.9)	17.6 (16.4-18.9)	55.0 (53.3-56.7)
Drink ever				
*No*	94 734	18.0 (17.4-18.7)	18.8 (18.2-19.5)	61.3 (60.3-62.2)
*Yes*	46 282	14.7 (13.9-15.5)	16.1 (15.3-16.9)	53.8 (52.6-55.0)
Country level§				
Level of hospital beds per 1000 people				
*Quantile 1*	84 704	12.2 (11.6-12.7)	10.0 (9.5-10.4)	47.8 (46.6-49.0)
*Quantile 2*	25 170	19.4 (18.7-20.2)	22.6 (21.8-23.4)	63.4 (62.3-64.4)
*Quantile 3*	12 381	11.7 (10.7-12.8)	8.0 (7.3-8.8)	47.8 (45.8-49.7)
*Quantile 4*	18 761	10.8 (10.0-11.6)	9.4 (8.8-10.1)	51.2 (49.7-52.6)
Level of people using safely managed sanitation services (% of population)				
*Quantile 1*	73 140	19.5 (18.8-20.3)	22.4 (21.6-23.2)	63.6 (62.6-64.7)
*Quantile 2*	14 621	11.8 (11.0-12.7)	9.2 (8.4-10.0)	50.4 (48.9-52.0)
*Quantile 3*	14 699	8.6 (7.6-9.6)	7.1 (6.5-7.7)	41.6 (39.0-44.2)
*Quantile 4*	38 556	11.7 (11.1-12.2)	9.9 (9.4-10.4)	48.3 (47.3-49.3)
Level of rural population (% of total population)				
*Quantile 1*	19 547	9.8 (9.0-10.7)	7.8 (7.1-8.6)	39.7 (38.0-41.5)
*Quantile 2*	44 393	11.7 (11.3-12.2)	9.4 (9.0-9.8)	49.2 (48.1-50.2)
*Quantile 3*	13 005	9.4 (8.2-10.7)	8.3 (7.3-9.4)	43.9 (41.5-46.3)
*Quantile 4*	64 071	19.5 (18.8-20.3)	22.6 (21.8-23.4)	63.7 (62.7-64.8)
Level of current health expenditure (% of GDP)				
*Quantile 1*	75 361	19.5 (18.8-20.2)	22.2 (21.4-22.9)	63.6 (62.6-64.6)
*Quantile 2*	14 671	7.5 (6.7-8.4)	10.6 (9.5-11.8)	48.1 (45.4-50.8)
*Quantile 3*	21 035	8.1 (7.1-9.1)	6.7 (6.1-7.3)	42.4 (39.9-45.1)
*Quantile 4*	29 949	12.4 (11.9-13)	9.7 (9.2-10.1)	48.0 (47.0-48.9)
Level of gross enrolment ratio for tertiary education (%)				
*Quantile 1*	72 632	19.5 (18.8-20.2)	22.4 (21.6-23.1)	63.5 (62.5-64.5)
*Quantile 2*	21 064	10.6 (9.8-11.5)	7.9 (7.3-8.5)	46.9 (45.3-48.4)
*Quantile 3*	15 067	12.0 (11.0-13.2)	8.3 (7.5-9.1)	48.3 (46.6-50.1)
*Quantile 4*	32 253	11.0 (10.4-11.5)	10.0 (9.6-10.5)	47.5 (46.1-48.8)
Level of GDP				
*Quantile 1*	73 811	19.4 (18.7-20.1)	22.3 (21.5-23)	63.5 (62.5-64.5)
*Quantile 2*	13 694	12.7 (11.6-13.8)	10.7 (9.7-11.8)	51.8 (50.0-53.6)
*Quantile 3*	24 902	8.7 (8.0-9.4)	7.1 (6.6-7.6)	44.2 (42.2-46.3)
*Quantile 4*	28 609	12.5 (11.9-13.1)	10.1 (9.6-10.6)	47.8 (46.9-48.8)

ADL – activities of daily living, IADL – instrumental activity of daily living, GDP – gross domestic product, CI – confidence interval

*Data are represented as percentage (95% CI), unless otherwise indicated. All values were weighted and standardised to represent the total population of each country.

†The measurement of mobility disability was not available in KLoSA. Thus, estimated prevalence did not include participants from Korea.

‡Income level of participants was categorised by quintile of the annual per capita total household income based on country group, including individual and spouse earnings, capital income, income from an employer or private pension and annuity; income from public pensions; other government transfers; other regular payments.

§All country level data were taken from World Bank. Besides, these variables were categorised into four levels by quantile of these variables based on their numerical value. (quantile 1 (the lowest) to quantile 4 (the highest)).

### Prevalence of physical functional disability by country

**Figure 1:**
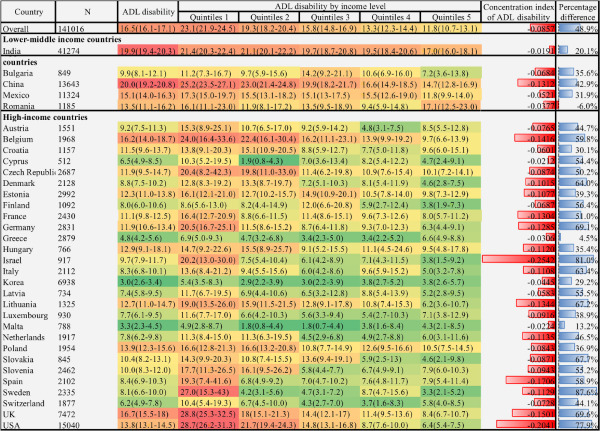
Heatmap of ADL disability prevalence and income-related inequality among adults aged 55 years or older: data from 33 countries, 2017-2020. The prevalence is expressed in heatmap fashion. Red indicates higher prevalence; green indicates lower prevalence. The concentration index is expressed in bar plot at the right side of heatmap, higher red bar indicates higher health inequality in the disadvantage income groups. Besides, the percentage difference is next to the concentration index, with higher bule bar indicates disability prevalence to be higher among quintile 1 groups than quintile 5 groups. Data are represented as percentage 95% CIs unless otherwise indicated. All values were weighted and standardised to represent the total population of each country. Countries were grouped into income groups according to the World Bank’s income classification. Percentage difference = ((quintile 1 − quintile 5)/ quintile 1) × 100%. ADL – activities of daily living.

**Figure 2:**
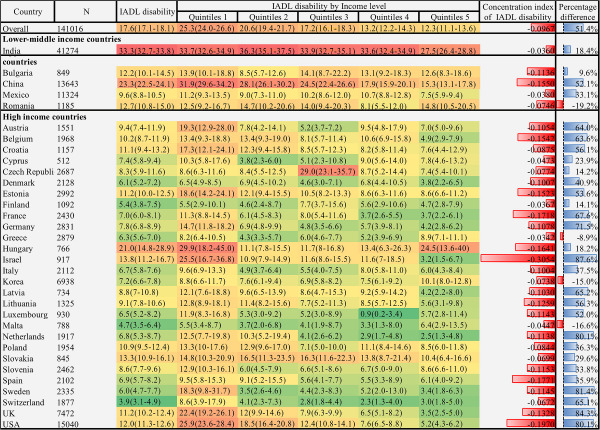
Heatmap of IADL disability prevalence and income-related inequality among adults aged 55 years or older: data from 33 countries, 2017-2020. The prevalence is expressed in heatmap fashion. Red indicates higher prevalence; green indicates lower prevalence. The concentration index is expressed in bar plot at the right side of heatmap, higher red bar indicates higher health inequality in the disadvantage income groups. Besides, the percentage difference is next to the concentration index, with higher bule bar indicates disability prevalence to be higher among quintile 1 groups than quintile 5 groups. Data are represented as percentage 95% CIs unless otherwise indicated. All values were weighted and standardised to represent the total population of each country. Countries were grouped into income groups according to the World Bank’s income classification. Percentage difference = ((quintile 1 − quintile 5)/ quintile 1) × 100%. IADL – instrumental activity of daily living.

**Figure 3:**
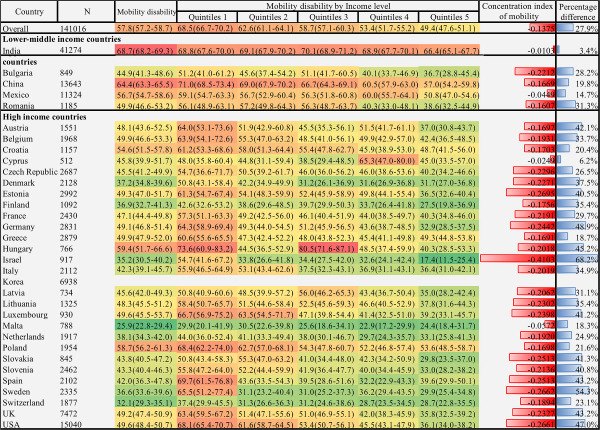
Heatmap of mobility disability prevalence and income-related inequality among adults aged 55 years or older: data from 33 countries, 2017-2020. The prevalence is expressed in heatmap fashion. Red indicates higher prevalence; green indicates lower prevalence. The concentration index is expressed in bar plot at the right side of heatmap, higher red bar indicates higher health inequality in the disadvantage income groups. Besides, the percentage difference is next to the concentration index, with higher bule bar indicates disability prevalence to be higher among quintile 1 groups than quintile 5 groups. Data are represented as percentage 95% CI unless otherwise indicated. All values were weighted and standardised to represent the total population of each country. Countries were grouped into income groups according to the World Bank’s income classification. Percentage difference = ((quintile 1 − quintile 5)/ quintile 1) × 100%.The measurement of mobility disability was not available in KLoSA. Thus, estimation did not include participants from Korea.

We found significant cross-national variation in physical functional disability, as presented in **Figure 1**, **Figure 2**, and **Figure 3**. The lowest prevalence of ADL disability was in Korea (3.0%; 95% CI = 2.6-3.4), followed by Malta (3.3%; 95% CI = 2.3-4.5) and Greece (4.8%; 95% CI = 4.2-5.6). The highest prevalence was in China (20%; 95% CI = 19.2-20.8) followed by India (19.9%; 95% CI = 19.4-20.3) and the UK (16.7%; 95% CI = 15.5-18.0). The cross-national variation in IADL and mobility disability prevalence was also substantial. The lowest prevalence of IADL disability was in Switzerland (3.9%; 95% CI = 3.1-4.9), followed by Malta (4.7%; 95% CI = 3.5-6.4) and Finland (5.4%; 95% CI = 3.8-7.5); the highest was in India (33.3%; 95% CI = 32.7, 33.8), followed by China (23.3%; 95% CI = 22.5-24.1) and Hungary (21.0%; 95% CI = 14.8, 28.9). Similarly, the lowest prevalence of mobility disability was in Malta (25.9%; 95% CI = 22.8-29.4), followed by Switzerland (32.1%; 95% CI = 29.3, 35.1) and Israel (35.2%; 95% CI = 30.5-40.2). The highest prevalence was in India 68.7% (95% CI = 68.2-69.3), followed by China 64.4% (95% CI = 63.3-65.5) and Hungary 59.4% (95% CI = 51.7-66.6).

### Income-related inequality of physical functional disability by country

**Figure 1**, **Figure 2**, and **Figure 3** showed substantial variation in physical functional disability prevalence within countries with respect to income level. Overall, the lowest income level group had the highest ADL disability prevalence (23.1%; 95% CI = 21.9-24.5), IADL disability (25.3%; 95% CI = 24.0-26.6), and mobility disability (68.5%; 95% CI = 66.7-70.2), while the highest income level group had the lowest ADL disability prevalence (11.8%; 95% CI = 10.7-13.1), IADL disability (12.3%; 95% CI = 11.1-13.6), and mobility disability (49.4%; 95% CI = 47.6-51.1). Concerning cross-national variation, the disability prevalence of the poorest quintile of the participants in high-income countries was similar to the disability prevalence of the upper-income participants from the lower-middle-income country.

Regarding the income-related inequality index of physical function disability, we estimated the overall concentration index value of ADL disability (-0.0857), IADL disability (-0.0967), and mobility disability (-0.1375), respectively. The result was consistent with the previous section, which indicated a pro-poor inequality of physical functional disability; low-income groups tended to have disabilities more than the high-income groups. Considerable variations of the concentration index value of all three domains of disability existed across these countries. The concentration index value of ADL disability was relatively higher in Israel (-0.2542), the USA (-0.2041), and Spain (-0.1706), yet modest in India (-0.0191), Cyprus (-0.0212), and Malta (-0.0224). Similarly, Israel (-0.3054), the USA (-0.1970), and Spain (-0.1771) showed higher inequality of IADL disability, while Greece (-0.0342), India (-0.0360), and Malta (-0.0367) showed lower inequality of IADL disability. Besides, the inequality of mobility function was also higher in Israel (-0.4103), Estonia (-0.2695), and Sweden (-0.2662) and lower in India (-0.0102), Cyprus (-0.0249), and Mexico (-0.0449). The concentration index value was higher in the high-income country group than in the lower-middle-income country group. The result of the percentage difference also followed the same patterns (**Figure 1**, **Figure 2**, and **Figure 3**).

### Decomposition of income-related health inequality

The RIF decomposition estimates of explanatory variables on income-related health inequality of physical functional disability are presented in **Table 2**. The potential explanatory variables for the inequality varied for the three functional disability indicators (ADL, IADL, and mobility function).

**Table 2:** RIF decomposition estimates of explanatory variables on income-related health inequality*

Variables		ADL disability			IADL disability			Mobility disability†	
**Individual level**	Coef (SE)		*P*-value	Coef (SE)		*P*-value	Coef (SE)		*P*-value
Age (ref = ≥55)									
*≥60*	0.032 (0.007)		<0.05	0.026 (0.008)		<0.05	0.050 (0.010)		<0.05
*≥65*	0.050 (0.007)		<0.05	0.040 (0.008)		<0.05	0.070 (0.010)		<0.05
*≥70*	0.057 (0.008)		<0.05	0.061 (0.008)		<0.05	0.100 (0.011)		<0.05
*≥75*	0.083 (0.009)		<0.05	0.079 (0.009)		<0.05	0.111 (0.012)		<0.05
*≥80*	0.058 (0.010)		<0.05	0.054 (0.010)		<0.05	0.103 (0.013)		<0.05
*≥85*	-0.098 (0.011)		<0.05	-0.159 (0.012)		<0.05	-0.007 (0.015)		>0.05
Gender (female vs male)	0.009 (0.005)		>0.05	0.005 (0.005)		>0.05	0.027 (0.007)		<0.05
Marital status (other vs married)	0.002 (0.005)		>0.05	-0.033 (0.005)		<0.05	-0.009 (0.007)		>0.05
Education (ref = less than upper secondary)									
Upper secondary and vocational training	0.017 (0.006)		<0.05	0.014 (0.006)		<0.05	0.021 (0.008)		<0.05
Tertiary	-0.026 (0.008)		<0.05	-0.072 (0.008)		<0.05	-0.113 (0.010)		<0.05
Chronic diseases (no vs yes)	0.005 (0.005)		>0.05	0.021 (0.006)		<0.05	0.072 (0.007)		<0.05
Current smoke (no vs yes)	-0.006 (0.007)		>0.05	0.006 (0.006)		>0.05	0.026 (0.009)		<0.05
Drink (no vs yes)	0.023 (0.006)		<0.05	0.009 (0.006)		>0.05	-0.007 (0.007)		>0.05
Country level‡									
Level of hospital beds per 1000 people (ref = Quantile 1)									
*Quantile 2*	0.057 (0.021)		<0.05	0.056 (0.024)		<0.05	0.033 (0.028)		>0.05
*Quantile 3*	0.025 (0.025)		>0.05	0.002 (0.028)		>0.05	-0.085 (0.033)		<0.05
*Quantile 4*	0.040 (0.026)		>0.05	0.012 (0.030)		>0.05	-0.069 (0.034)		<0.05
Level of people using safely managed sanitation services (% of population) (ref. = Quantile 1)									
*Quantile 2*	-0.013 (0.031)		>0.05	0.020 (0.035)		>0.05	0.009 (0.040)		>0.05
*Quantile 3*	-0.036 (0.032)		>0.05	-0.038 (0.036)		>0.05	-0.071 (0.043)		>0.05
*Quantile 4*	-0.051 (0.037)		>0.05	-0.029 (0.041)		>0.05	-0.062 (0.049)		>0.05
Level of Rural population (% of total population) (ref. = Quantile 1)			>0.05						
*Quantile 2*	0.009 (0.021)		>0.05	0.018 (0.023)		>0.05	0.001 (0.029)		>0.05
*Quantile 3*	0.018 (0.022)		>0.05	0.025 (0.024)		>0.05	0.025 (0.028)		>0.05
*Quantile 4*	0.020 (0.028)		>0.05	0.030 (0.031)		>0.05	0.049 (0.037)		>0.05
Level of Current health expenditure (% of GDP) (ref = Quantile 1)			>0.05						
*Quantile 2*	-0.013 (0.020)		>0.05	-0.027 (0.022)		>0.05	-0.044 (0.027)		>0.05
*Quantile 3*	-0.022 (0.034)		>0.05	-0.032 (0.038)		>0.05	-0.116 (0.051)		<0.05
*Quantile 4*	-0.077 (0.036)		<0.05	-0.108 (0.040)		<0.05	-0.103 (0.053)		>0.05
Level of Gross enrolment ratio for tertiary education (%) (ref. = Quantile 1)									
*Quantile 2*	-0.007 (0.024)		>0.05	0.008 (0.027)		>0.05	-0.039 (0.032)		>0.05
*Quantile 3*	-0.006 (0.030)		>0.05	0.017 (0.033)		>0.05	0.029 (0.040)		>0.05
*Quantile 4*	-0.008 (0.021)		>0.05	-0.007 (0.023)		>0.05	-0.027 (0.029)		>0.05
Level of GDP (ref = Quantile 1)			>0.05			>0.05			>0.05
*Quantile 2*	-0.042 (0.029)		>0.05	-0.036 (0.032)		>0.05	-0.058 (0.038)		>0.05
*Quantile 3*	0.028 (0.032)		>0.05	0.051 (0.036)		>0.05	0.096 (0.049)		>0.05
*Quantile 4*	0.049 (0.044)		>0.05	0.092 (0.049)		>0.05	0.059 (0.058)		>0.05

RIF – recentred influence functions, ADL – activities of daily living, IADL – instrumental activity of daily living, coef – coefficients, SE – standard error, ref – reference group, GDP – gross domestic product

*Data are represented as coef (SE), unless otherwise indicated.

†The measurement of mobility disability was not available in KLoSA, so the estimated prevalence did not include participants from Korea.

‡All country level data were taken from World Bank. Besides, these variables were categorised into four levels by quantile of these variables based on their numerical value (quantile 1 (the lowest) to quantile 4 (the highest)).

For ADL disability inequality, the individual level variables, including age, and drinking behaviour, were positively correlated with health inequality of ADL disability, while education was negatively correlated with health inequality. The country-level variable, like the higher level of health expenditure, was negatively correlated with health inequality, while the lower level of hospital beds per 1000 people was positively correlated with health inequality. For IADL disability inequality, individual-level variables like age and chronic diseases were positively correlated with health inequality of IADL disability, while married and higher education levels were negatively correlated with health inequality. The country-level variables, like the higher level of health expenditure, were negatively correlated with health inequality, while the lower level of hospital beds per 1000 people was positively correlated with health inequality. Lastly, for mobility disability inequality, the individual level variables like age, female, chronic diseases, and smoking behaviour were positively correlated with health inequality of mobility disability, while higher education level was negatively correlated with health inequality. The country-level variable, like the higher level of hospital beds per 1000 people and higher level of health expenditure, were negatively correlated with health inequality.

## DISCUSSION

We found that the disability prevalence of ADL, IADL, and mobility function was higher in lower-middle-income countries and more prevalent in the poor-income groups in all study countries. The concentration index value showed a substantial difference in health inequality, while health inequality was higher in high-income countries. Regarding the decomposition of disability inequality, we found that individual married, tertiary education, and country-level health infrastructure and resources were associated with reduced health inequality. However, age, unhealthy lifestyles, and chronic diseases were associated with increased health inequality.

We found substantial variations in physical functional disability across countries, while disability prevalence was higher in lower-middle-income countries. A previous study using the World Health Survey 2002-2004 data containing 49 countries also found that the age-standardised disability prevalence among adults aged 18 and above was higher in low and lower-middle-income countries [6]; these disabilities were grouped into eight domains: vision, mobility, self-care, cognition, interpersonal activities, pain and discomfort, sleep and energy, and affect. Our study added to the current literature by further focusing on the physical functional capacity of individuals and using validated measurement tools, including ADL, IADL, and mobility, thus increasing the robustness of these comparisons and providing new evidence in older adult populations.

The negative concentration index value in ADL, IADL, and mobility function showed that the lower-income groups tended to have higher disability prevalence than higher-income groups, and this pro-poor inequality in disability was consistent across countries. Our findings were consistent with previous studies which found physical functional disability was more prevalent among the poor income groups and the same in most populations worldwide [7-9]. We also found substantial variations between countries in the magnitude of inequalities, with higher inequalities in high-income countries. A trend study in the USA and UK populations found that recent cohorts are ageing better, but only among the wealthiest groups [33]; another study conducted in Europe also found that socioeconomic inequalities in disability appear to have increased over time between 2002 and 2017 [34]. One reason could be that higher-income countries often have lower social transfers and privately grounded health systems, which could reduce the accessibility to health care resources for social disadvantages [35]. A previous study also found publicly-funded health care was associated with increased longevity, and reducing inequalities in social determinants is essential to promoting healthy ageing in populations worldwide [36]. These findings indicate that inequality in physical functional disability can be complex and severe.

Various determinants might drive the cross-national variability in the inequality of physical functional disability. We decomposed the income-related inequalities of disability into individual and country levels to better understand the causes of income-related inequalities in disability and provide evidence for effective prevention and interventions. These findings were consistent with previous studies that individual age, married, education [10], unhealthy lifestyles [37], and chronic diseases [15] affect disability. Besides, our study also complements existing literature by consolidating the current evidence with harmonised measurements and investigating the contribution of these risk factors to income-related inequalities in disability across countries, and by exploring how country-level contextual factors shape individual health inequality. We found that the country-level health infrastructure and resources [15], like the higher level of hospital beds per 1000 people and higher level of health expenditure, were associated with reduced health inequality. This is in line with a previous study, which found that social spending positively impacts reducing health inequality in Europe[38].

The ageing of populations worldwide makes healthy ageing key to helping older adults maintain their health as long as possible and reducing the societal burden associated with it. A previous study found that low educational levels and wealth may have deteriorated health conditions in early life stages, resulting in persistent health inequality in old age [39]. Besides, disadvantages accumulated over the life course could make older adults who are poor more vulnerable in health status and have more significant needs for health care resources [40]. In line with our findings, higher health infrastructure and resources could help older adults access appropriate, affordable, quality health care and thus help to improve healthy ageing.

### Strengths and limitations

This study has several strengths. First, we included a large-scale, representative sample from harmonised data across 33 countries and used the same instruments and methodology to collect functional physical health and other information at the individual level, providing a more comprehensive and robust measurement. Second, we extended the age of participants to middle-aged as mid-life is the critical period of physical function impairment onset in older adults. We also decomposed the household income-related inequality in disability into individual and country-level contributing factors using this large data set, providing comprehensive insights into multilevel determinants of inequality in disability.

This study also has some limitations. First, the association of individual and country-level factors with income-related health inequality was based on the cross-sectional design, which limits the interpretation of our findings. Thus, future studies could examine this association using a cohort study design. Second, other potential factors that might need to be considered in the decomposition analysis. Finally, the study countries were selected based on the convenience of secondary data and may not be representative of the global population or countries with similar social-economic development; caution is warranted when generalising the findings beyond this scope. Nonetheless, the large sample size and unified methodology provide valuable insights into the prevalence and inequality of physical functional disability across a diverse range of countries, making our estimates a reasonable proxy for real circumstances.

## CONCLUSIONS

We measured and compared the cross-national variation in the prevalence and inequality of physical functional disability and investigated the potential determinants of income-related inequality among middle-aged and older adults aged 55 and older from 33 countries. We found that physical functional disability was higher in lower-middle-income countries and more prevalent in the low-income groups. Income-related inequality in disability existed in all countries, while the magnitude of inequality was higher in high-income countries. Inequality in disability was associated with individual lifestyles and country-specific social-economic and health care determinants. Therefore, future research and policies must identify the factors contributing to inequality from both individual and contextual levels to narrow the inequality of physical functional disability.

## Additional material


Online Supplementary Document

## References

[1] World Health Organization. World report on disability 2011. Geneva: World Health Organization; 2011.

[2] United Nations. World population ageing 2020 highlights. 2020. New York: United Nations; 2020.

[3] Prince MJ Wu F Guo Y Gutierrez Robledo LM O’Donnell M Sullivan R The burden of disease in older people and implications for health policy and practice. Lancet. 2015;385:549-62. 10.1016/S0140-6736(14)61347-7 25468153

[4] Chatterji S Byles J Cutler D Seeman T Verdes E Health, functioning, and disability in older adults–present status and future implications. Lancet. 2015;385:563-75. 10.1016/S0140-6736(14)61462-8 25468158 PMC4882096

[5] United Nations. Ageing and disability. 2015. Available: https://www.un.org/development/desa/disabilities/disability-and-ageing.html. Accessed: 16 May 2023.

[6] Hosseinpoor AR Stewart Williams JA Gautam J Posarac A Officer A Verdes E Socioeconomic inequality in disability among adults: a multicountry study using the World Health Survey. Am J Public Health. 2013;103:1278-86. 10.2105/AJPH.2012.301115 23678901 PMC3682610

[7] Stefler D Prina M Wu YT Sanchez-Niubo A Lu W Haro JM Socioeconomic inequalities in physical and cognitive functioning: cross-sectional evidence from 37 cohorts across 28 countries in the ATHLOS project. J Epidemiol Community Health. 2021;75:980-6. 10.1136/jech-2020-214714 33649052

[8] Makaroun LK Brown RT Diaz-Ramirez LG Ahalt C Boscardin WJ Lang-Brown S Wealth-Associated Disparities in Death and Disability in the United States and England. JAMA Intern Med. 2017;177:1745-53. 10.1001/jamainternmed.2017.3903 29059279 PMC5820733

[9] von dem Knesebeck O Vonneilich N Ludecke D Income and functional limitations among the aged in Europe: a trend analysis in 16 countries. J Epidemiol Community Health. 2017;71:584-91. 10.1136/jech-2016-208369 28062642

[10] Hosseinpoor AR Bergen N Kostanjsek N Kowal P Officer A Chatterji S Socio-demographic patterns of disability among older adult populations of low-income and middle-income countries: results from World Health Survey. Int J Public Health. 2016;61:337-45. 10.1007/s00038-015-0742-3 26537634 PMC4879166

[11] Amilon A Hansen KM Kjær AA Steffensen T Estimating disability prevalence and disability-related inequalities: Does the choice of measure matter? Soc Sci Med. 2021;272:113740. 10.1016/j.socscimed.2021.113740 33571943

[12] World Health Organization. IFC: International Classification of Functioning, Disability and Health. 2001. Available: https://www.who.int/standards/classifications/international-classification-of-functioning-disability-and-health. Accessed: 16 May 2023.

[13] Patel R Srivastava S Kumar P Chauhan S Govindu MD Jean Simon D Socio-economic inequality in functional disability and impairments with focus on instrumental activity of daily living: a study on older adults in India. BMC Public Health. 2021;21:1541. 10.1186/s12889-021-11591-1 34384409 PMC8359266

[14] Zhong Y Wang J Nicholas S Gender, childhood and adult socioeconomic inequalities in functional disability among Chinese older adults. Int J Equity Health. 2017;16:165. 10.1186/s12939-017-0662-3 28865465 PMC5581446

[15] Zhang T Liu C Lu B Wang X Changes of inequality in functional disability of older populations in China from 2008 to 2018: a decomposition analysis. BMC Geriatr. 2022;22:308. 10.1186/s12877-022-02987-8 35397500 PMC8994264

[16] Cambois E Sole-Auro A Bronnum-Hansen H Egidi V Jagger C Jeune B Educational differentials in disability vary across and within welfare regimes: a comparison of 26 European countries in 2009. J Epidemiol Community Health. 2016;70:331-8. 10.1136/jech-2015-205978 26546286

[17] Clarke P Smith J Aging in a cultural context: cross-national differences in disability and the moderating role of personal control among older adults in the United States and England. J Gerontol B Psychol Sci Soc Sci. 2011;66:457-67. 10.1093/geronb/gbr054 21666145 PMC3132269

[18] Sonnega A Faul JD Ofstedal MB Langa KM Phillips JW Weir DR Cohort Profile: the Health and Retirement Study (HRS). Int J Epidemiol. 2014;43:576-85. 10.1093/ije/dyu067 24671021 PMC3997380

[19] Steptoe A Breeze E Banks J Nazroo J Cohort profile: the English longitudinal study of ageing. Int J Epidemiol. 2013;42:1640-8. 10.1093/ije/dys168 23143611 PMC3900867

[20] Börsch-Supan A Brandt M Hunkler C Kneip T Korbmacher J Malter F Data Resource Profile: the Survey of Health, Ageing and Retirement in Europe (SHARE). Int J Epidemiol. 2013;42:992-1001. 10.1093/ije/dyt088 23778574 PMC3780997

[21] Jang SN Kawachi I Chang J Boo K Shin HG Lee H Marital status, gender, and depression: analysis of the baseline survey of the Korean Longitudinal Study of Ageing (KLoSA). Soc Sci Med. 2009;69:1608-15. 10.1016/j.socscimed.2009.09.007 19819601

[22] Perianayagam A Bloom D Lee J Parasuraman S Sekher TV Mohanty SK Cohort Profile: The Longitudinal Ageing Study in India (LASI). Int J Epidemiol. 2022;51:e167-76. 10.1093/ije/dyab266 35021187 PMC9365624

[23] Wong R Michaels-Obregon A Palloni A Cohort Profile: The Mexican Health and Aging Study (MHAS). Int J Epidemiol. 2017;46:e2. 10.1093/ije/dyu263 25626437 PMC5837398

[24] Zhao Y Hu Y Smith JP Strauss J Yang G Cohort profile: the China Health and Retirement Longitudinal Study (CHARLS). Int J Epidemiol. 2014;43:61-8. 10.1093/ije/dys203 23243115 PMC3937970

[25] Lee J Phillips D Wilkens J Gateway to Global Aging Data Team Gateway to Global Aging Data: Resources for Cross-National Comparisons of Family, Social Environment, and Healthy Aging. J Gerontol B Psychol Sci Soc Sci. 2021;76 Suppl 1:S5-16. 10.1093/geronb/gbab050 33861849 PMC8186854

[26] The World Bank. World development report 2017: Governance and the Law. Washington, DC:The World Bank; 2017.

[27] Arel-Bundock V. WDI: World Development Indicators and Other World Bank Data. R package version 2.7.6. 2022. Available: https://CRAN.R-project.org/package=WDI. Accessed: 16 May 2023.

[28] Galobardes B Shaw M Lawlor DA Lynch JW Davey Smith G Indicators of socioeconomic position (part 1). J Epidemiol Community Health. 2006;60:7-12. 10.1136/jech.2004.023531 16361448 PMC2465546

[29] United Nations. World Population Prospects 2019. New York: United Nations; 2019.

[30] Wagstaff A Paci P van Doorslaer E On the measurement of inequalities in health. Soc Sci Med. 1991;33:545-57. 10.1016/0277-9536(91)90212-U 1962226

[31] Erreygers G Correcting the concentration index. J Health Econ. 2009;28:504-15. 10.1016/j.jhealeco.2008.02.003 18367273

[32] Heckley G Gerdtham UG Kjellsson G A general method for decomposing the causes of socioeconomic inequality in health. J Health Econ. 2016;48:89-106. 10.1016/j.jhealeco.2016.03.006 27137844

[33] de la Fuente J Caballero FF Verdes E Rodriguez-Artalejo F Cabello M de la Torre-Luque A Are younger cohorts in the USA and England ageing better? Int J Epidemiol. 2019;48:1906-13. 10.1093/ije/dyz126 31873752 PMC6929538

[34] Rubio Valverde JR Mackenbach JP Nusselder WJ Trends in inequalities in disability in Europe between 2002 and 2017. J Epidemiol Community Health. 2021;75:712-20. 10.1136/jech-2020-216141 33674458 PMC8292565

[35] Brennenstuhl S Quesnel-Vallee A McDonough P Welfare regimes, population health and health inequalities: a research synthesis. J Epidemiol Community Health. 2012;66:397-409. 10.1136/jech-2011-200277 22080814

[36] Galvani-Townsend S Martinez I Pandey A Is life expectancy higher in countries and territories with publicly funded health care? Global analysis of health care access and the social determinants of health. J Glob Health. 2022;12:04091. 10.7189/jogh.12.04091 36370409 PMC9653205

[37] Stansfeld SA Head J Fuhrer R Wardle J Cattell V Social inequalities in depressive symptoms and physical functioning in the Whitehall II study: exploring a common cause explanation. J Epidemiol Community Health. 2003;57:361-7. 10.1136/jech.57.5.361 12700221 PMC1732450

[38] Álvarez-Gálvez J Jaime-Castillo AM The impact of social expenditure on health inequalities in Europe. Soc Sci Med. 2018;200:9-18. 10.1016/j.socscimed.2018.01.006 29355829

[39] Wu YT Daskalopoulou C Muniz Terrera G Sanchez Niubo A Rodriguez-Artalejo F Ayuso-Mateos JL Education and wealth inequalities in healthy ageing in eight harmonised cohorts in the ATHLOS consortium: a population-based study. Lancet Public Health. 2020;5:e386-94. 10.1016/S2468-2667(20)30077-3 32619540 PMC7739372

[40] United Nations. Health Inequalities in Old Age. 2018. Available: https://www.un.org/development/desa/ageing/news/2018/04/health-inequalities-in-old-age/. Accessed: 16 May 2023.

