# Supplementary material for: Cross-country comparison of income-related inequality in physical functional disability among middle-aged and older adults: Evidence from 33 countries
Source: J Glob Health. 2023 May 19;13:04053. doi: 10.7189/jogh.13.04053 (PMC10197408; doi:10.7189/jogh.13.04053)
Supplement: Online Supplementary Document [file jogh-13-04053-s001.pdf]

Table S1 specific questionnaires of physical functional health measures

Domains	Items	Description of Questionnaires
ADL	Bathing	Because of a health or memory problem do you have any difficulty with bathing or showering?
	Dressing	Because of a health or memory problem do you have any difficulty with dressing,including putting on shoes and socks?
	Eating	Because of a health or memory problem do you have any difficulty with eating, such as cutting up your food?
	Getting in/out of bed	Because of a health or memory problem do you have any difficulty with getting in or out of bed?
	Using the toilet	Because of a health or memory problem do you have any difficulty with using the toilet, including getting up and down?
IADL	Managing money	Because of a health or memory problem do you have any difficulty with managing your money -- such as paying your bills and keeping track of expenses
	Taking medications	Because of a health or memory problem do you have) any difficulty taking medications?
	Shopping for groceries	Because of a health or memory problem do you have any difficulty with shopping for groceries?
	Preparing hot meals	Because of a health or memory problem do you have any difficulty preparing a hot meal?
Mobility functions	Walking one block or 100 yards/meters	Because of a health problem do you have any difficulty with walking one block or 100 yards/meters?
	Climbing several flights of stairs	Because of a health problem do you have any difficulty with climbing several flights of stairs without resting?
	Getting up from a chair after sitting for long periods	Because of a health problem do you have any difficulty with getting up from a chair after sitting for long periods?
	Stooping kneeling or crouching	Because of a health problem do you have any difficulty with stooping, kneeling, or crouching?
	Reaching or extending arms above shoulder level	Because of a health problem do you have any difficulty with reaching or extending your arms above shoulder level?
	Lifting or carrying objects weighting over 5 kg or 10 pounds	Because of a health problem do you have any difficulty with lifting or carrying weights over 5 kg or 10 pounds, like a heavy bag of groceries?
	Picking up a small coin from the table activities	Because of a health problem do you have any difficulty with picking up a dime from a table?

Note: All of the surveys collected information on physical functional health, but varied in the spectrum and details of questions asked. Therefore, to facilitate cross-country comparisons across the studies, this study utilize a binary scale (Yes/No) to maximize comparability across all surveys. Technical details of the harmonized process could refer to ‘Codi Young, Youngha Oh, Sandy Chien, Peifeng Hu. Harmonization of cross-national studies of aging to the Health and Retirement Study - user guide: Physical Functional Health. University of Southern California, CESR-Schaeffer Working Paper Series No. 2022-001. Published December 2021."

Table S2 Description of country-level varibales

Country-level varibales	Description
Hospital beds (per 1,000 people)	Hospital beds include inpatient beds available in public, private, general, and specialized hospitals and rehabilitation centers. In most cases beds for both acute and chronic care are included.
People using safely managed sanitation services (% of population)	The percentage of people using improved sanitation facilities that are not shared with other households and where excreta are safely disposed of in situ or transported and treated offsite. Improved sanitation facilities include flush/pour flush to piped sewer systems, septic tanks or pit latrines: ventilated improved pit latrines, compositing toilets or pit latrines with slabs.
Rural population (% of total population)	Rural population refers to people living in rural areas as defined by national statistical offices. It is calculated as the difference between total population and urban population.
Current health expenditure (% of GDP)	Level of current health expenditure expressed as a percentage of GDP. Estimates of current health expenditures include healthcare goods and services consumed during each year. This indicator does not include capital health expenditures such as buildings, machinery, IT and stocks of vaccines for emergency or outbreaks.
Gross enrolment ratio for tertiary education, both sexes (%)	Gross enrollment ratio is the ratio of total enrollment, regardless of age, to the population of the age group that officially corresponds to the level of education shown. Tertiary education, whether or not to an advanced research qualification, normally requires, as a minimum condition of admission, the successful completion of education at the secondary level.
GDP, PPP (constant 2017 international $)	PPP GDP is gross domestic product converted to international dollars using purchasing power parity rates. An international dollar has the same purchasing power over GDP as the U.S. dollar has in the United States. GDP is the sum of gross value added by all resident producers in the country plus any product taxes and minus any subsidies not included in the value of the products. It is calculated without making deductions for depreciation of fabricated assets or for depletion and degradation of natural resources. Data are in constant 2017 international dollars.
